# Therapeutic routine with respiratory exercises improves posture, muscle activity, and respiratory pattern of patients with neck pain: a randomized controlled trial

**DOI:** 10.1038/s41598-022-08128-w

**Published:** 2022-03-09

**Authors:** Hamid Rezaee Dareh-deh, Malihe Hadadnezhad, Amir Letafatkar, Anneli Peolsson

**Affiliations:** 1 Faculty of Physical Education and Sports Sciences, Kharazmi University, Tehran, Iran; 2 Sports Injury and Corrective Exercises, Kharazmi University, Tehran, Iran; 3 Department of Health, Medicine and Caring Sciences, Unit of Physiotherapy, Linköping University, Lasarettsgatan, house 511, 14th floor, Campus US, 58183 Linköping, Sweden

## Abstract

Neck pain and forward head posture (FHP) are typical in prolonged smartphone users and need to be targeted for treatment. We aimed to compare the effect of a routine therapeutic program with and without respiratory exercises on smartphone users with FHP and non-specific chronic neck pain (NSCNP). Sixty patients (aged 24.7 ± 2.1 years) with FHP and NSCNP were randomly assigned to the routine therapeutic program (n = 20), combined respiratory exercises with a routine therapeutic program (n = 20), or control (n = 20) groups. At baseline, there was no difference among groups at all variables. Each programme was implemented three times a week for eight weeks. Primary Outcome was pain measured by visual analogue scale (VAS), and secondary ones were forward head angle, the activity of specific muscles, and respiratory patterns, measured by photogrammetry, electromyography and manual, respectively. All outcomes were measured at baseline and eight weeks post-treatment. We used the repeated measures analysis of variance to examine the interaction between time and group, paired t-test for intragroup comparison, one-way analysis of variance for intergroup comparison, and Tukey post hoc test at a significant level 95% was used. There were significant differences in the combined group compared with the routine therapeutic group (*P* = 0.03) for diaphragm muscle activation, respiratory balance (*P* = 0.01), and the number of breaths (*P* = 0.02). There were significant within-group changes from baseline to post-treatment in the combined group for all outcomes above, but no changes in the therapeutic exercise routine group. Despite respiratory pattern, none of the secondary outcomes proved to be superior in the combination group compared to the routine therapeutic program in smartphone users with FHP and NSCNP. Future studies with longer follow-up assessments could strengthen these results.

*Trial registration*: Current Controlled Trials using the IRCT website with ID number of, IRCT20200212046469N1 “Prospectively registered” at 04/03/2020.

## Introduction

The use of electronic tools is increasing worldwide^1^. Varieties and the attractiveness of these tools have led to various groups of people, especially teenagers, using them for prolonged periods resulted in some musculoskeletal problems^2^.

It is commonly thought that in addition to psychological problems, like anxiety, headaches, insomnia, depression, poor sleep quality, and fatigue, long-term use of smartphones leads to inactivity and abnormal posture, such as a forward head posture (FHP) and rounded shoulders^3^. Maintaining a forward head posture (FHP) decreases cervical lordosis of the lower cervical vertebrae and creates a posterior curve in the upper thoracic vertebrae to maintain balance. This can necessarily affect muscular activity and place more pressure on the cervical spine where chronic pain originates^4,5^.

Individuals with FHP and non-specific chronic neck pain (NSCNP) often suffer from weakness of the deep neck flexor muscles, which is to be compensated by excessive activity of other muscles such as sternocleidomastoid, and scalene^6^. This compensation leads to muscle imbalance and changes in the stress–strain diagram, by which cervical spine overload occurs^6,7^. This may also be observed in the thoracic spine as some involved muscles are connected to both areas. Also, disorders or impairments occur in the neck and respiratory system because of the joint activity of the muscles mentioned above operated on the neck movements and respiratory function^6,8^.

Maximal inspiratory and expiratory pressure (MIP & MEP) decrease in individuals with FHP and NSCNP^8^. This posture causes the respiratory pattern to change from nasal breathing to mouth breathing. Compared to the normal posture, both scalene and sternocleidomastoid muscles show a higher activity amount in FHP^6^. Such long-term activation can create poor respiratory habits to facilitate activities in auxiliary respiratory muscles^4^. Furthermore, the respiratory function is affected by changed muscle activity due to pain and disability, affecting the neck in a vicious circle. In order to improve this posture, heat, traction, and exercise have all been used^7^. Various methods such as joint mobilization, stretching, isometric strengthening exercises, endurance exercises, and proprioceptive exercises have also been applied depending on the method and theory utilized by the therapist or the patient’s condition^7^ Also, various therapeutic and rehabilitative methods have been used in some previous research, such as the McKenzie exercise, Kinesio taping, and myofascial release^9^. Each method has demonstrated positive results in improving impairments and disorders in this area^9^. In this regard, some researchers have reported better consequences obtained from combinations of some of their therapeutic methods^9^. However, previous research has not compared the effect of adding breathing exercises to therapeutic exercises; also, respiratory exercises have shown benefits for respiration and balancing the main and auxiliary muscles^6^. Furthermore, respiratory exercises are low-cost and easily used in different situations^6^.

This study investigated if the addition of respiratory exercise for smartphone users with FHD and NSCNP, into a routine therapeutic program, would produce superior results than the routine therapeutic program itself? It was hypothesized that adding respiratory exercise into a routine therapeutic would enhance treatment effects on neck pain, respiratory pattern, electromyography, and posture in smartphone users with moderate chronic neck pain and FHP.

## Methods

### Study design and participants

The study was a randomised assessor-blind controlled trial. The study has been registered at the Iranian Registry of Clinical Trials prospectively registered at 04/03/2020 (IRCT20200212046469N1), and the Ethics Committee on Research obtained the ethical approval at the Kharazmi University, Iran (IR.KHU.REC.1398.023). The study was conducted at the Laboratory of Biomechanics and Sports Injuries Department, Kharazmi University, Tehran, Iran. The study was reported following the rigor of the CONSORT guideline^10^, and all experimental conditions conformed to the Declaration of Helsinki. Orthopaedic physicians recruited patients with chronic neck pain via flyers displayed at the hospitals over three months from April to June 2020.

Prior to participation in the study, all subjects were explained about the objectives and provided written informed consent, and all participants provided written informed consent prior to enrolment.

Inclusion criteria were males and females who were using a smartphone for more than four hours a day who rated their ‘worst pain over the last 24-h’ as moderate using the visual analogue scale (VAS), with neck disability index (NDI) scores between 28 and 45%, and pain lasting longer than three months^11–14^. FHP was defined as a cervical angle < 50°^15–17^. A lateral-view photograph was taken to identify cervical angle in standing position^15–17^.

The exclusion criteria were: previous history of neck or back surgery, neurological signs, rheumatoid arthritis, and currently using muscle relaxation medication.

**Figure 1:**
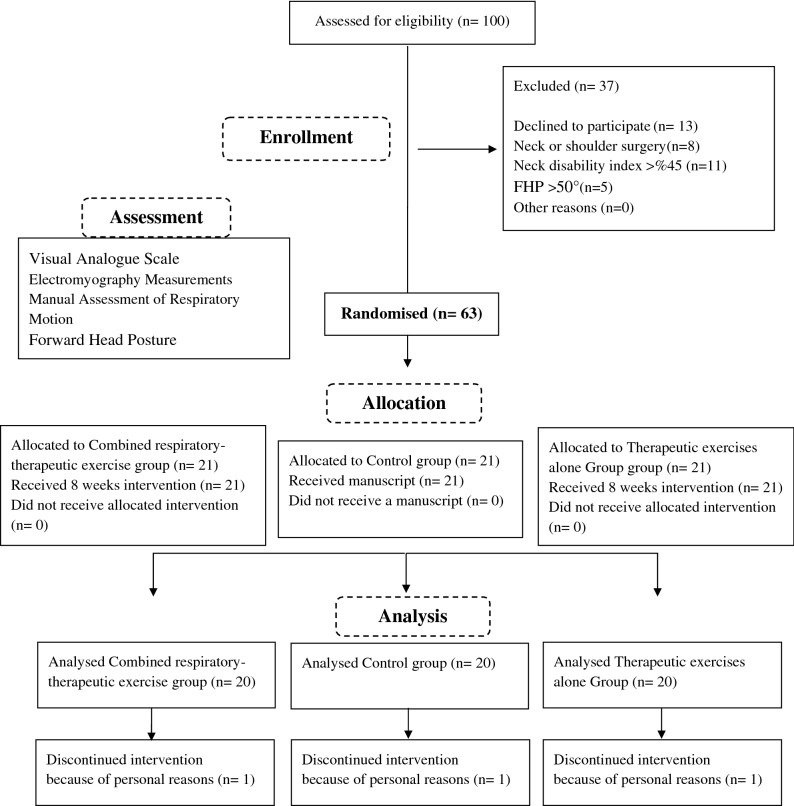
Flow Diagram of the study.

Participants were randomised into two experimental groups and one control group by drawing a number from 1 to 63, placed in sealed envelopes in a box prepared in advance by the trainer. The randomisation sequence was not disclosed until participants had completed their baseline assessments. The assessor was blinded to group allocation. Participants were not blinded to the exercise study; however, they were not aware which treatment was considered to be therapeutic. The same physiotherapist and trainer supervised both active treatment groups (Fig. 1).

### Outcome assessments

The outcomes were measured at baseline and—for organisational reasons of the Laboratory -post-treatment two days after the eight-week intervention. The primary outcome measure was pain, and secondary outcome measures were EMG, respiratory pattern, and posture.

All participants completed a baseline questionnaire (see Table 1). A PhD trained physiotherapist performed a physical therapy evaluation with 25-years of clinical experience. All participants were instructed to limit their weekly exercise to the study treatment^5,18,19^.

**Table 1:** Demographic data and baseline values of patients.

Characteristic	Control Group	Combined respiratory-therapeutic exercise group	Therapeutic exercises alone Group	*P* value
Age, y	25.3 ± 1.4	23.9 ± 2.3	24.9 ± 2.8	0.35
Height, m	177.9 ± 4.5	177.8 ± 5.4	177.0 ± 5.7	0.90
Weight, kg	72.8 ± 4.9	71.8 ± 6.0	72.2 ± 4.2	0.90
Body mass index, kg/m^2^	23.0 ± 1.4	22.6 ± 1.1	23.8 ± 1.2	0.62
FHP, Degree	45.3 ± 3.7	46.5 ± 2.3	47.5 ± 4.1	0.24
Pain, VAS	4.8 ± 0.9	5.1 ± 1.6	4.0 ± 1.0	0.97
NDI, score	38.9% ± 3.7	40.2% ± 3.9	39.5% ± 4.1	0.33
Smartphone use, h	4.1 ± 1.7	4.0 ± 2.1	4.2 ± 1.9	0.56

*VAS* Visual analogue scale, *FHP* forward head posture, *NDI* neck disability index.

#### Pain intensity

The pain was evaluated using VAS choosing a number from 0 (no pain at all) to 10 (unbearable pain). This scale is a valid and reliable tool^19–21^. The minimum clinically important difference for within-group on the pain scale has been reported to be 2.5-points in people with chronic neck pain^22^.

#### Electromyography

An EMG device with eight channels (made by data Log Biometrics company, Canada) was used to measure the activity of upper trapezius muscles, sternocleidomastoid, scalene, neck erector spine, and diaphragm muscles. The muscles reported above were chosen because directly involved in the functional activities of the cervical-thoracic district (e.g., neck movements, trunk control, and breathing)^23^. Based on the recommendations of SENIAM, the skin surface was shaved of hair and cleaned with alcohol swabs before wireless EMG electrodes were applied.

EMG electrodes were placed in five areas as follows: upper trapezius, as positioned from the lateral to the midpoint, as an imaginary line was formed by the posterior aspect of the acromion and the spinous process of C7, and the electrode was placed on the muscle bulk^24^. For the sternocleidomastoid, the electrode was placed at the lower one-third of the line connecting the sternal notch and mastoid process^24^. For scalene muscles, the electrode was placed on the posterior triangle of the scalene muscle, above the clavicle, more inclined to the sternocleidomastoid (just posterior to and at a slightly oblique angle relative to the sternocleidomastoid [SCM], just above the clavicle and in the hollow triangle anterior of the upper trapezius)^25^. For neck erector spine, the electrode was attached to the muscles around the C4 vertebra^26^. For the diaphragm, the lower edge of the rib cage on a vertical line that passes through the nipple centre was selected for electrode placement^27,28^.

The EMG information was collected using an EMG device with a sampling frequency of 1000 Hz, and in this study the EMG signal data were sampled at 1000-Hz. These signals were filtered in the bandpass between 20 and 500 Hz^24^. The full shoulder flexion task was used to obtain data on the activity of the selected muscles. In this regard, the subjects performed each flexion movement and returned to the initial state at a 5-s time, in three consecutive times^24,25,29^.

Additionally, to estimate a maximum voluntary contraction (MVC) for the upper trapezius (ICC = 0.88), subjects placed their hand at 90^0^ abduction, sitting down on a chair, and were asked to apply pressure against the exposed resistance at the top^29,30^. To obtain the MVC for sternocleidomastoid (ICC = 0.97) and scalene (ICC = 0.87) muscles, subjects were placed in the supine position, and their hands were put on their own heads. Then, the head was anterolaterally placed and pressurised against the hand resistance^25^. To obtain the MVC for the erector spine muscle (ICC = 0.87–0.95), the subjects were asked to be in a prone posture and put both hands behind their head as moving the overhand against the resistance in the extension direction^30^. To achieve MVC for the diaphragm muscle in the sitting position subjects took deep breaths^31^. Each position of the maximal voluntary contraction was used two times for a five-second duration to normalise the data^30^.

The EMG signal was processed by the Root Mean Square (RMS) algorithm in the MATLAB program. The resulting number represented the average power of a signal that indicated the muscle activity. To compare the subjects and normalise the data, the obtained values from the RMS were divided by those obtained from the MVC of each muscle, and the amount of muscle activity was considered as a percentage of the MVC^24,25,29^.

#### Respiratory pattern assessment

**Figure 2:**
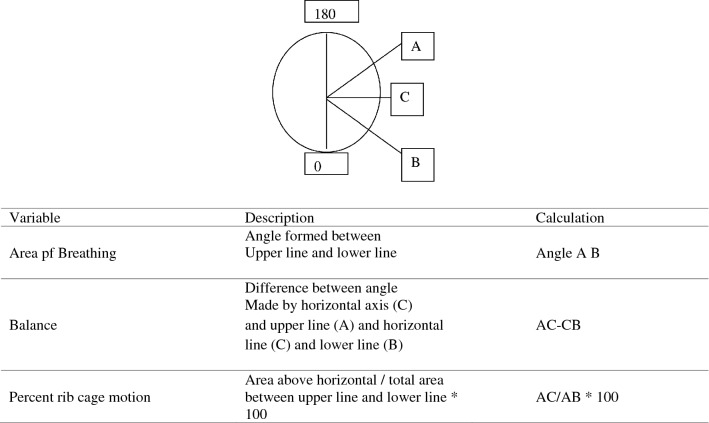
The MARM graphic notation.

**Figure 3:**
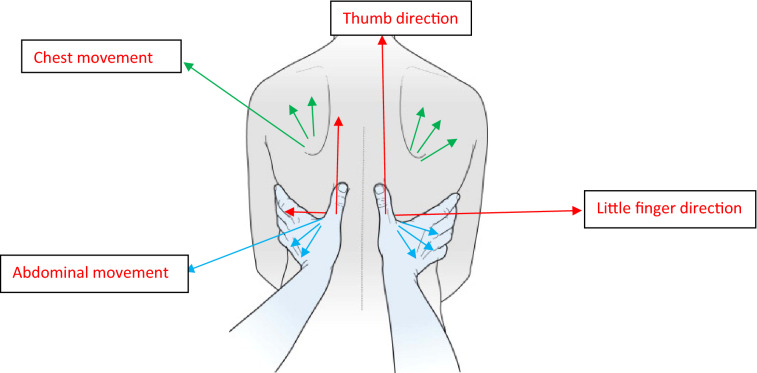
MARM test.

To assess the breathing pattern, the Manual Assessment of Respiratory Motion (MARM) was adopted presenting a good reliability (ICC = 0.850)^18^ (Fig. 2). Sit behind the subject and place both your hands on the lower lateral rib cage so that your whole hand rests firmly and comfortably and does not restrict breathing motion. Your thumbs should be approximately parallel to the spine, pointing vertically, and your hand comfortably open with fingers spread so that the little finger approaches a horizontal orientation. Note that the fourth and fifth fingers reach below the lower ribs and can feel the abdominal expansion. You will make an assessment of the extent of overall vertical motion your hands feel relative to the overall lateral motion. Also, decide if the motion is predominantly upper rib cage, lower rib cage/abdomen or relatively balanced. Use this information to determine the relative distance from the horizontal line (C) of the upper and lower lines of the MARM diagram. The upper line (A) will be further from the horizontal and closer to the top if there is more vertical and upper rib cage motion. The lower line (B) will be further from the horizontal and closer to the bottom if there is more lateral and lower rib cage/abdomen motion^18^ (Fig. 3). Finally, get a sense of the overall magnitude and freedom of rib cage motion. Place lines further apart to represent greater overall motion and closer for less motion. the tester recorded and interpreted different aspects of respiration, including the number of breaths and the balance of respiration between the upper and lower parts of the rib cage and abdomen. In this way, in the pre-test and post-test, after evaluating the respiratory status with this method and determining the ratio of the share of respiration in the abdominal and thoracic parts, the changes in the respiratory share of these two parts are analyzed and the results are obtained. The indicator shows the effectiveness of the interventions applied^18^.

#### Forward head posture assessment

The forward head and shoulder angles were measured using photogrammetry of the sagittal plane. This method favoured reliability (ICC ranged from 0.88 to 0.98), and it has been used in various research^4,25,32,33^. Forward head posture was defined as a cervical angle < 50°^15–17^. A lateral-view photograph was taken to identify cervical angles in a standing position^15–17^. Three anatomical signs, including left tragus, acromion, and the spinous process of C7-vertebra were determined and marked to measure the angles. Then, the subjects were asked to stand at the designated area beside a wall (at a 23-cm distance) so that their left side was placed toward the wall. The photographic tripod supporting the digital camera was placed at a distance of 265-cm, and its height was set based on the subject’s right shoulder level. In such circumstances, the subjects were asked to lean forward three times and raise their hand over their head three times. They were then asked to stand in a completely relaxed and natural posture, and to look at an imaginary point on the opposite wall (eyes in line with horizon). The tester took images of the body profile view after a five-second pause. Finally, these images were transferred to a computer, and the angle of the line connecting tragus to C7-vertebrae, and that of the line connecting C7 and the acromion process were respectively measured with the vertical line (forward head and shoulder angles) using Kinova software (Kinova-0.8.27-64-bit, Kinova company, Canada)^34,35^.

### Training protocol

As intended in this study, training included two parts: therapeutic routine and respiratory exercises. The therapeutic exercises contained resistance and stretching exercises (in the three stretching exercises, we used static stretching with a 30-s hold for 2-sets) for 45 to 60 min per session, specifically one session a day for three sessions a week; totally all held in eight weeks^36–40^. The rest interval between movements in these exercises was 45 and 30 s for resistance and stretching exercises, respectively. Resistance exercises included: 1.Side-lying external rotation(Teres minor, infraspinatus), 2.Prone horizontal abduction with external, rotation(Middle trapezius, Lower trapezius, Rhomboids, Infraspinatus, Teres minor), 3.Y-to-I exercise(Middle trapezius, Lower trapezius, Serratus anterior): Subjects try to flex the shoulder 180 degrees while externally rotating while in the prone position with the shoulder at a 90-degree abduction., 4.Chin tuck (Longus colli, Longus capitis): Subjects bring the chin close to the chest while lying on the supine position., and stretching exercises included: 1. Static levator scapulae stretch (levator scapulae) exercise(Pectorals minor), 2.One-sided unilateral self-stretch exercise (Pectorals minor): Subjects stands back against the wall at a distance, and while placing one forearm on the wall, the body rotates in the opposite direction. , 3.Static sternocleidomastoid stretch (Sternocleidomastoid)^37^. In the combined group, respiratory exercises were added to the therapeutic routine above, which consisted of balloon breathing exercises performed in sessions of four sets: The subject lies in the supine position, placing the soles of his feet against the wall so that the ankle, knee, and thigh joints are at a 90-degree angle. The subject places a 3–4-inch ball between his/her knees, which he/she has to maintain through the pressure of the internal thigh muscles during the whole training period and puts his/her back on the bed through a flat pelvic tilt. Holds the right hand above the head and the left hand with the balloon. It inhales through the nose in three-four seconds and then exhales slowly into the balloon. To perform the next tail operation, place only the tongue on the roof of the mouth without biting the balloon to prevent air from escaping inside the balloon., and as each set had four complete breathing breaks, these exercises were conducted for two sessions a day and three days a week for eight weeks^41^. All exercise was done under the supervision of a physical therapist at the pain clinic. All participants received documentation, including information on postural corrections and improving general health.

### Control group

The control group (n = 20) received a pamphlet including information on postural corrections and improving general health during the 8-week study period. No other physical therapy modalities or treatments were performed^42^.

### Statistical analysis

The necessary sample size was estimated using G*Power 3.1.7 for Windows (G*Power©, University of Dusseldorf, Germany). To obtain 80% statistical power, an α error = 0.05, repeated-measure analysis of variance (ANOVA), and a medium effect size of 0.25 to consider two groups and two measurements for the primary outcome (neck pain), generating a sample size about of 18-participants per group (total sample size of 54-subjects) considering a 15%-dropout rate the sample was increased to 63 (21 in each group).

One-way ANOVA was used to compare the group demographics. The analysis of covariance (ANCOVA), with a between-factor of the groups and participants' baseline scores included as a covariate^43^. The Bryant-Paulson procedure was used to conduct pairwise comparisons and calculate the confidence intervals^44^. Effect sizes of 0.2, 0.5, and 0.8 were considered ‘small’, ‘moderate’, and ‘large’ respectively^41^. SPSS software was (alpha level of 0.05) used for statistical analysis (IBM Corp., Armonk, NY, USA).

## Results

One hundred participants were recruited, 37 did not qualify based on the exclusion and inclusion criteria of the study, and 63 were randomised into three groups. Intention to treat protocol was followed for post-treatment analysis. See the CONSORT diagram for details.

There was a high degree of adherence to all three interventions. Of the possible 24-sessions, the therapeutic routine group, the combined group, and the control group attended 20 ± 2 sessions, 20 ± 1 sessions, and 19 ± 2 sessions, respectively.

Our cohort comprised male and female subjects, with a mean age of 24.7 ± 2.1 years, with 4.6 ± 1.1 pain on VAS at baseline and 46.4 ± 3.4 on the FHP. Further details on demographic data and additional baseline outcome measures are reported in Table 1. Demographic characteristics and baseline outcome measures (age (*P* = 0.35), pain (*P* = 0.97), and FHP (*P* = 0.24)) did not differ between the groups (*P* > 0.05).

### Treatment effects

The main effects of group × time interactions are presented in Tables 2, 3, and 4.

**Table 2:** Effect of training on pain and forward head posture.

Within-group							Between-groups		
Outcomes	Groups	Baseline Mean ± SD	8-weeks Mean ± SD	Change relative to baseline^c^ (%)	D^†^ and 95% CI (Lower limit -Upper limit)	*P*	Interaction effects		*P*
Pain, VAS	Combined respiratory-therapeutic exercise	5.1 ± 1.6	1.9 ± 0.6^d^	62.4% ↓	0.85 (0.41 to 1.33)	0.01	F = 3.22 P = 0.04^e^		0.01^a^ 0.01^b^
	Therapeutic exercise alone	4.0 ± 1.0	1.7 ± 0.8^d^	57.5% ↓	0.75 (0.23 to 1.14)	0.02			
	Control	4.8 ± 0.9	4.5 ± 0.4	6.2% ↓	0.21 (− 1.2 to 0.03)	0.38			
FHP, Degree	Combined respiratory-therapeutic exercise	46.5 ± 2.3	52.8 ± 3.9^d^	13.5% ↑	− 0.70 (− 2.5 to − 0.38)	0.01	F = 5.32 P = 0.03^e^		0.01^a^ 0.02^b^
	Therapeutic exercise alone	47.5 ± 4.1	52.3 ± 2.5^d^	10.1% ↑	− 0.55 (− 1.92 to − 0.13)	0.03			
	Control	45.3 ± 3.7	46.9 ± 4.8	3.5% ↑	− 0.12 (− 0.08 to 1.08)	0.43			

*VAS* visual analogue scale, *FHP* forward head posture.

^a^Significant between combined respiratory-therapeutic exercise and control groups.

^b^Significant between therapeutic exercise alone and control groups.

^c^Percent change (↓decrease, ↑increase).

^d^Denotes significant within group improvement between the baseline and 8-weeks treatment period.

^e^Significant group × time interaction.

**Table 3:** Effect of training on muscle activation (%MVC).

Within-group							Between-groups	
Outcomes	Groups	Baseline Mean ± SD	8-weeks Mean ± SD	Change Relative to Baseline^c^ (%)	D^†^ and 95% CI (Lower limit -Upper limit)	*P*	Interaction effects	*P*
Upper trapezius	Combined respiratory-therapeutic exercise	14.6 ± 1.8	5.8 ± 0.9	60.3% ↓	0.95 (0.41 to 1.33)	0.01^d^	F = 7.42 P = 0.04f.	0.02^a^ 0.03^b^
	Therapeutic exercise alone	11.2 ± 2.3	6.1 ± 1.1	45.5% ↓	0.81 (0.17 to 1.12)	0.02^d^		
	Control	12.5 ± 1.4	10.4 ± 2.5	16.8% ↓	0.21 (− 1.2 to 0.14)	0.13		
Sternocleidomastoid	Combined respiratory-therapeutic exercise	68.1 ± 5.8	43.4 ± 5.0	36.3% ↓	0.91 (0.5 to 2.42)	0.01^d^	F = 13.84 P = 0.02f.	0.02^a^ 0.03^b^
	Therapeutic exercise alone	63.2 ± 7.5	44.7 ± 6.5	29.3% ↓	0.79 (0.23 to 1.44)	0.03^d^		
	Control	66.5 ± 7.1	64.7 ± 6.9	2.7% ↓	0.05 (− 0.08 to 1.48)	0.16		
Scalene	Combined respiratory-therapeutic exercise	38.6 ± 3.7	21.0 ± 3.3	45.6% ↓	0.92 (0.41 to 2.14)	0.01^d^	F = 14.48 P = 0.02f.	0.01^a^ 0.02^b^
	Therapeutic exercise alone	33.8 ± 4.0	20.5 ± 3.6	39.3% ↓	0.86 (0.13 to 1.62)	0.02^d^		
	Control	35.4 ± 3.1	33.8 ± 2.8	4.5% ↓	0.26 (− 0.4 to 0.10)	0.21		
Neck erector spinae	Combined respiratory-therapeutic exercise	28.8 ± 3.7	14.5 ± 3.0	49.6% ↓	0.90 (0.12 to 1.94)	0.01^d^	F = 11.24 P = 0.03f.	0.02^a^ 0.03^b^
	Therapeutic exercise alone	23.7 ± 2.8	15.9 ± 1.9	32.9% ↓	0.85 (0.17 to 1.64)	0.04^d^		
	Control	25.2 ± 3.1	26.9 ± 2.8	6.7% ↑	− 0.27 (− 0.22 to 0.8)	0.16		
Diaphragm	Combined respiratory-therapeutic exercise	58.5 ± 7.2	75.0 ± 5.5	28.2% ↑	− 0.78 (− 0.2 to − 0.98)	0.01^d^	F = 14.12 P = 0.03f.	0.01^a^ 0.03^e^
	Therapeutic exercise alone	61.1 ± 6.7	58.4 ± 7.9	4.4% ↓	0.18 (− 0.2 to 0.46)	0.11		
	Control	55.4 ± 5.3	53.8 ± 6.4	2.9% ↓	0.13 (− 0.12 to 0.07)	0.22		

^a^Significant between combined respiratory-therapeutic exercise and control groups.

^b^Significant between therapeutic exercise alone and control groups.

^c^Percent change (↓decrease, ↑increase).

^d^Denotes significant within group improvement between the baseline and 8-weeks treatment period.

^e^Significant between combined respiratory-therapeutic exercise and therapeutic exercise alone groups.

^f^Significant group × time interaction.

**Table 4:** Effect of training on respiratory pattern.

Within-group							Between-groups		
Outcomes	Groups	Baseline Mean ± SD	8-weeks Mean ± SD	Change Relative to Baseline^c^ (%)	D^†^ and 95% CI (Lower limit -Upper limit)	*P*	Interaction Effects		*P*
Respiratory balance	Combined respiratory-therapeutic exercise	26.9 ± 7.3	37.0 ± 6.2	37.5% ↑	− 0.59 (− 0.12 to − 1.33)	0.01^d^	F = 12.6 P = 0.03^e^		0.01^a^ 0.02^b^
	Therapeutic exercise alone	28.6 ± 8.2	30.5 ± 9.2	6.6% ↑	− 0.10 (− 0.09 to 0.78)	0.08			
	Control	30.0 ± 9.4	28.8 ± 9.1	4% ↓	0.06 (− 1.2 to 0.03)	0.18			
Number of breaths, n/min	Combined respiratory-therapeutic exercise	22.5 ± 2.3	18.2 ± 1.7	19.1% ↓	0.72 (0.21 to 1.37)	0.02^d^	F = 8.7 P = 0.03^e^		0.01^a^ 0.02^b^
	Therapeutic exercise alone	23.5 ± 3.1	21.4 ± 2.2	8.9% ↓	0.36 (− 0.31 to 0.28)	0.06			
	Control	19.8 ± 1.8	20.0 ± 1.5	1% ↑	− 0.06 (− 0.08 to 0.44)	0.43			

^a^Significant between combined respiratory-therapeutic exercise and control groups.

^b^Significant between combined respiratory-therapeutic exercise and therapeutic exercise alone groups.

^c^Percent change (↓decrease, ↑increase).

^d^Denotes significant within group improvement between the baseline and 8-weeks treatment period.

^e^Significant group × time interaction.

Significant groups by time interactions were found for pain (F = 3.22, *P* = 0.04), FHP (F = 5.32, *P* = 0.03), amount of activity in the upper trapezius muscle (F = 7.42, *P* = 0.04), amount of activity in the sternocleidomastoid muscle (F = 13.84, *P* = 0.02), amount of activity in the scalene muscle (F = 14.48, *P* = 0.02), for the amount of activity in the neck erector spine muscle (F = 11.24, *P* = 0.03), amount of activity in the neck erector diaphragm muscle (F = 14.12, *P* = 0.03), respiratory balance (F = 12.6, *P* = 0.03), number of breaths (F = 8.7, *P* = 0.03).

For pain, at eight weeks both the therapeutic routine (57.5% changes from the baseline), (d(95%CI) = 0.75 (0.23, 1.14); *P* = 0.02) and combined group (62.4% changes from the baseline), (D(95%CI) = 0.85 (0.41, 1.33); *P* = 0.01) had significant within-group changes, but differences in the control group (6.2% changes from the baseline), (d(95% CI) = 0.21 (− 1.2, 0.03); *P* = 0.38) were not significant.

For FHP, at eight weeks both the therapeutic routine (47.5°–52.3°), (d(95% CI) = (− 0.55 (− 1.92, − 0.13); *P* = 0.03) and combined groups (46.5°–52.8°), (d(95%CI) = (− 0.70 (− 2.5, − 0.38); *P* = 0.01) had significant within-group changes, but differences in control group (45.3°–46.9°), (d(95%CI) = (− 0.12 (− 0.08, 1.08) *P* = 0.43) were not significant (see in Table 2).

For amount of activity muscles, at eight weeks the therapeutic routine groups (upper trapezius (45.5% changes from the baseline), (d(95%CI) = (0.81 (0.17, 1.12); *P* = 0.02), sternocleidomastoid (29.3% changes from the baseline), (d(95%CI) = (0.79 (0.23, 1.44)); *P* = 0.03), scalene (39.3% changes from the baseline), (d(95%CI) = (0.86 (0.13, 1.62)); *P* = 0.02), and neck erector spine (32.9% changes from the baseline), (d(95%CI) = (0.85 (0.17, 1.64)); *P* = 0.04), and in combined groups (upper trapezius (60.3% changes from the baseline), (d(95%CI) = (0.95 (0.41, 1.33)); *P* = 0.01), sternocleidomastoid (36.3% changes from the baseline), (d(95%CI) = (0.91 (0.5, 2.42)); *P* = 0.01), scalene (45.6% changes from the baseline), (d(95%CI) = (0.92 (0.41, 2.14)); *P* = 0.01), neck erector spine (49.6% changes from the baseline), (d(95%CI) = (0.90 (0.12, 1.94)); *P* = 0.01), and diaphragm (28.2% changes from the baseline), (d(95%CI) = (− 0.78 (− 0.2, − 0.98)); *P* = 0.01)) groups had significant within-group changes, but differences in control group and amount of activity diaphragm muscle in the therapeutic routine group (4.4% changes from the baseline), (d(95%CI) = (0.18 (− 0.2, 0.46)); *P* = 0.11) were not significant (see in Table 3).

For respiratory pattern, at eight weeks both the combined group (respiratory balance (37.5% changes from the baseline), (d(95%CI) = (− 0.59 (− 0.12, − 1.33)); *P* = 0.01), and number of breaths (19.1% changes from the baseline), (d(95%CI) = (0.72 (0.21, 1.37)); *P* = 0.02), but differences in the control group, and therapeutic routine were not significant (see in Table 4).

## Discussion

In this randomized, assessor-blind controlled trial, we found evidence that in smartphone users with FHP and NSCNP, additional respiratory exercises during a therapeutic program had no extra benefit on pain intensity, forward head angle and muscle activity directly after the 8-week intervention compared to the same therapeutic program without respiratory exercises. Furthermore, despite respiratory pattern, none of the secondary outcomes proved superior in the combination group.

Moreover, the amount of activity in the upper trapezius, sternocleidomastoid, scalene, and cervical erector spine muscles revealed a significant decrease in both experimental groups, although no significant differences were found between these two groups. Having conducted the training interventions, the amount of activity in the diaphragm muscle indicated a remarkable increase in the combined group as compared with the two other groups, including the therapeutic and control ones. As for the respiratory pattern, superiority was observed in the number of breaths and respiratory balance for the group of combined exercises compared to the therapeutic routine and control groups. In the control group, there were no significant differences over time.

Having performed the intended exercises, a decrease in activity could be found in the upper trapezius, sternocleidomastoid, scalene, and cervical erector spinae muscles in both exercise groups. However, changes in the diaphragm muscle activity were only observed in the combined group.

Suggested that performing breathing exercises is effective in improving function for patients with FHP^6^. This study suggested that SCM and anterior scalene activities increased in both groups when comparing the changes within groups. However, respiratory feedback exercises more effectively induced activity changes in the SCM when comparing changes between groups. Excessive tension and contraction of neck muscles happen by compensation for patients with FHP^6^. This leads to decreased frequency of contraction and relaxation of muscles as muscle activities due to the stiffness of neck flexors increase. However, inhibition of compensation is effective if a proper load is applied during inhalation and exhalation using respiratory feedback exercises^6^. Based on this study, when breathing exercises are mediated, they effectively release the body. Exercises are thought to effectively improve the NDI, which is a subjective functional scale. This study suggests that is more efficient in SCM activity, neck flexor, and NDI by mediating respiratory feedback exercise than those in control group. Such results can affect inefficient breathing imbalances of patients with FHP, as neck flexors are accessory respiratory muscles^6^.

Regarding muscle activity, the present results are consistent with Lee et al. and Borisut et al.^45,46^. As the electronic tool being used in the FHP, recent studies have indicated that increased activity was observed in the muscles, including the upper trapezius, sternocleidomastoid, cervical erector spinae, thoracic erector spinae, and neck extensors. Consequently, these muscles are shortened, and the deep cervical flexor muscles are weakened in such conditions^47^. In this regard, Borisut et al. reported a decrease in the muscle activation of the cervical erector spineae, sternocleidomastoid, anterior scalene, and upper trapezius after strength exercises^46^.

Having performed all the exercises in both experimental groups in the present study, the probable effect mechanism can be implied as activation in the collaborative muscles in the cervical area, correcting FHP, and decreasing the activity amount in muscles, including the upper trapezius, sternocleidomastoid, scalene, and cervical erector spineae^46^. In fact, adding the respiratory exercises to the therapeutic ones did not create a remarkable change in the activity of the upper trapezius, sternocleidomastoid, scalene, and cervical erector spineae muscles. However, a significant increase in diaphragm muscle activity was revealed due to the respiratory exercises in the combined group compared with the two other groups.

As for the FHP, the experimental groups indicated a considerable decrease compared to the controls, but no significant difference was observed between the experimental groups. In this regard, the results obtained in this research were consistent with those found by Kong et al.^7^. According to the previous research, the chin-tuck exercise merely is not of enough durability^48^. Hence, researchers have tried to combine this exercise with some other endurance and strength exercises, to strengthen the movement domain and increase the endurance of the cervical muscles. Kong et al.^7^ reported that performing a course of modified cervical exercises has revealed a remarkable positive effect on the FHP of smartphone users who suffered from such an abnormal disorder. Therefore, the probable mechanism to decrease the FHP in both experimental groups has been assumed to reduce the activity of upper trapezius, sternocleidomastoid, scalene, and cervical erector spinae muscles, strengthen cervical deep flexor muscles, and use collaborative muscles in this area^7^.

Before and after the performance of the training interventions in the three involved groups, the number of pain changes were measured and evaluated using VAS. Although the controls experienced no significant reduction in pain, both experimental groups’ showed an observable decrease in pain following the interventions. Having reviewed the obtained results, no significant difference was found based on the effectiveness of pain between the two experimental groups.

As for the pain variable, the research results were consistent with those obtained from Chung et al.’s study^36^, in which two exercise methods of ‘craniocervical flexion and isometric neck exercise’ were compared in patients with chronic neck pain in terms of the effect on pain. Their comparative results indicated that both mentioned exercise methods have remarkably improved pain^36^. The present research aimed to compare the effect caused by adding respiratory exercises to the therapeutic routine by applying mere therapeutic exercises using a different method. Having conducted all the exercises, consequently, both experimental groups benefited from a remarkable decrease in their amount of pain while no significant difference was observed between both groups in this regard. As inferred from these findings, the addition of respiratory exercises did not increase the effect of the therapeutic ones on decreasing pain. Therefore, the positive effect of therapeutic exercises on balancing cervical muscle activity and improving posture in this area can be simply deemed the leading cause of pain reduction.

On the other hand, the respiratory pattern was evaluated using the MARM method in this research, and the obtained results revealed that only the combined group experienced positive changes in this pattern compared with the therapeutic routine. Generally, these results were consistent with those observed in Lee et al.’s study^1^. Having examined whether and how a course of exercises affects the cervical angle and respiratory function in smartphone users, Lee et al.^1^ reported that the participants conducted the related exercises, experienced positive and remarkable results in the cervical angle and multiple respiratory factors as compared with the controls^1^. As aimed in this research preface, the breath number and respiratory balance were compared to be affected by the respiratory-therapeutic and therapeutic exercises. The possible mechanism of effects caused by the respiratory-therapeutic exercises, which focused on correcting the FHP, seemed to strengthen respiratory muscles such as the diaphragm, increase lung volume, extend vital capacity and inform people with their body and breath position. Accordingly, the respiratory pattern was associated with positive changes in the respiratory-therapeutic exercise group^1,49^.

Our study limitation was that no long-term follow-up assessment was considered in the current study, so a similar study with a follow-up stage is highly recommended^38–40^. Future research should combine the assessment of neck impairments^50^ with advanced respiratory assessment and respiratory exercise tools such as spirometry and power breath to assess respiratory factors associated with neck pain. Finally, qualitative studies (e.g., focus group, interviews) should consider the patients’ perspectives (e.g., expectations, beliefs)^51^ regarding the respiratory exercises in neck pain to inform clinicians on their feasibility in the clinical settings.

## Conclusions

Besides improvement in main symptoms, if the purpose of treatment for patients with a forward head posture and chronic neck pain is to correct respiratory pattern, this study recommends adding respiratory exercise to a routine therapeutic program.

## Supplementary Information

Supplementary Information 1.

Supplementary Information 2.

Supplementary Information 3.

Supplementary Information 4.

## Abbreviations

FHP: Forward head posture
NSCNP: Non-specific chronic neck pain
MIP & MEP: Maximal inspiratory and expiratory pressure
EMG: Electromyography
VAS: Visual analogue scale
NDI: Neck disability index
MVC: Maximum voluntary contraction
SCM: Sternocleidomastoid
RMS: Root Mean Square
MARM: Manual Assessment of Respiratory Motion
ANOVA: Analysis of variance
ANCOVA: Analysis of covariance
CI95%: 95% Confidence intervals
d: Effect size

## References

[1] Lee NK, Jung SI, Lee DY, Kang KW (2017). Effects of exercise on cervical angle and respiratory function in smartphone users. Oso Pub. Heal. Rese. Persp..

[2] Jung SI, Lee NK, Kang KW, Kim K (2016). The effect of smartphone usage time on posture and respiratory function. Phys. Ther. Sci..

[3] Selvaganapathy K, Rajappan R, Dee TH (2017). the effect of smartphone addiction on craniovertebral angle and depression status among university students. Int. J. Integr. Med. Sci..

[4] Gadotti IC, Biasotto-Gonzalez DA (2010). Sensitivity of clinical assessments of sagittal head posture. J. Eval. Clin. Pract..

[5] Alshahrani A, Aly SM, Abdrabo MS, Asiri FY (2018). Impact of smartphone usage on cervical proprioception and balance in healthy adults. Biomed. Res..

[6] Kang J-I, Jeong D-K, Choi H (2016). The effect of feedback respiratory exercise on muscle activity, craniovertebral angle, and neck disability index of the neck flexors of patients with forward head posture. Phys. Ther. Sci..

[7] Kong YS, Kim YM, Shim J (2017). The effect of modified cervical exercise on smartphone users with forward head posture. Phys. Ther. Sci..

[8] Dimitriadis Z, Kapreli E, Strimpakos N, Oldham J (2013). Respiratory weakness in patients with chronic neck pain. Man. Ther..

[9] Kim J, Kim S, Shim J (2018). Effects of McKenzie exercise, Kinesio taping, and myofascial release on the forward head posture. Phys. Ther. Sci..

[10] Schulz KF, Altman DG, David Moher CONSORT (2010). Statement: Updated guidelines for reporting parallel group randomised trials. Trials J..

[11] Fejer R, Kyvik KO, Hartvigsen J (2006). The prevalence of neck pain in the world population: A systematic critical review of the literature. Eur. Spine J..

[12] Bovim G, Schrader H, Sand T (1994). Neck pain in the general population. Spine.

[13] Pr Blanpied, Ar Gross, Elliott J (2017). Neck pain: Revision 2017 clinical practice guidelines linked to the international classification of functioning, disability and health from the orthopaedic section of the american physical therapy association. J. Orthop. Sports Phys. Ther..

[14] Bartley EJ, Fillingim RB (2013). Sex differences in pain: A brief review of clinical and experimental findings. Br. J. Anaesth..

[15] Yip CH, Chiu TT, Poon AT (2008). The relationship between head posture and severity and disability of patients with neck pain. Man. Ther..

[16] Lee KJ, Han HY, Cheon SH (2015). The effect of forward head posture on muscle activity during neck protraction and retraction. Phys. Ther. Sci..

[17] De-la-Llave-Rincón AI, Fernández-de-las-Peñas C, Palacios-Ceña D (2009). Increased forward head posture and restricted cervical range of motion in patients with carpal tunnel syndrome. J. Orthop. Sports Phys. Ther..

[18] Courtney R, Van DÆJ (2008). Evaluation of breathing pattern: Comparison of a manual assessment of respiratory motion (MARM) and respiratory induction plethysmography. App. Psyc. Bio..

[19] Cleland JA, Childs JD, Whitman JM (2008). Psychometric properties of the Neck Disability Index and numeric pain rating scale in patients with mechanical neck pain. Arch. Phys. Med. Rehabil..

[20] Kim D, Cho M, Park Y (2015). Effect of an exercise program for posture correction on musculoskeletal pain. Phys. Ther. Sci..

[21] Jensen MP, Karoly P, Braver S (1986). The measurement of clinical pain intensity: A comparison of six methods. Pain.

[22] Kovacs FM, Abraira V, Royuela A (2008). Spanish back pain research network. Minimum detectable and minimal clinically important changes for pain in patients with nonspecific neck pain. BMC Musculoskelet Disord..

[23] Donald A. Neumann. Kinesiology of the musculoskeletal system. (2016).

[24] Jw K, Sm S, Nk L (2015). Changes in upper-extremity muscle activities due to head position in subjects with a forward head posture and rounded shoulders. Phys. Ther. Sci..

[25] Kim BB, Lee JH, Jeong HJ (2016). Effects of suboccipital release with Craniocervical flexion exercise on Craniocervical alignment and extrinsic cervical muscle activity in subjects with forward head posture. Electromyogr. Kinesiol..

[26] Article O (2016). Effect of duration of smartphone use on muscle fatigue and pain caused by forward head posture in adults. Phys. Ther. Sci..

[27] Dds SV, Dds RM, Ignacia M (2019). Awake teeth grinding in participants with canine guidance or group function: Effect on diaphragm EMG activity, heart rate, and oxygen saturation. J. Cra SLE Pra..

[28] Petersen E, Buchner H, Eger M, Rostalski P (2017). Convolutive blind source separation of surface EMG measurements of the respiratory muscles. Bio. Eng. Bio. Tec..

[29] Thigpen CA, Padua DA, Michener LA (2010). Head and shoulder posture affect scapular mechanics and muscle activity in overhead tasks. Electromyogr. Kinesiol..

[30] Chaikumarn M (2017). Repeatability of EMG normalization of the neck and shoulder muscles in symptomatic office workers. Int. J. Occ. Saf. Ergo..

[31] Walterspacher S, Pietsch F, Walker DJ, Röcker K, Kabitz HJ (2018). Activation of respiratory muscles during respiratory muscle training. Res. Phys. Neur..

[32] Ruivo RM, Carita AI, Pezarat-Correia P (2015). The effects of training and detraining after an 8-month resistance and stretching training program on forward head and protracted shoulder postures in adolescents: Randomised controlled study. Man. Ther..

[33] Richards KV, Beales DJ, Smith AJ, O'Sullivan PO, Straker LM (2016). Neck posture clusters and their association with biopsychosocial factors and neck pain in Australian adolescents. Phys. Ther..

[34] Shereen H, Elwardany WH, El-Sayed MF (2015). Reliability of Kinovea computer program in measuring cervical range of motion in sagittal plane. Open Access Lib. J..

[35] Harman K, Cheryl L, Kozey H, Butler H (2005). Effectiveness of an exercise program to improve forward head posture in normal adults: A randomized, controlled 10-week trial. Man. Ther..

[36] Chung S, Jeong YG (2018). Effects of the craniocervical flexion and isometric neck exercise compared in patients with chronic neck pain: A randomized controlled trial. Phys. Rev..

[37] Ruivo RM, Pezarat-correia P, Carita AI (2017). Effects of a resistance and stretching training program on forward head and protracted shoulder posture in adolescents. Manipul. Phys. Ther..

[38] Price J, Rushton A, Tyros V, Heneghan NR (2021). Expert consensus on the important chronic non-specific neck pain motor control and segmental exercise and dosage variables: An international e-Delphi study. PLoS One..

[39] Price J, Rushton A, Tyros I, Tyros V, Heneghan NR (2020). Effectiveness and optimal dosage of exercise training for chronic non-specific neck pain: A systematic review with a narrative synthesis. PLoS One..

[40] O'Riordan C, Clifford A, Van De Ven P, Nelson J (2014). Chronic neck pain and exercise interventions: Frequency, intensity, time, and type principle. Arch. Phys. Med. Rehabil..

[41] Boyle KL, Olinick J, Lewis C (2010). The value of blowing up a balloon. North Am. J. Sports Phys. Ther..

[42] Im B, Kim Y, Chung Y, Hwang S (2016). Effects of scapular stabilization exercise on neck posture and muscle activation in individuals with neck pain and forward head posture. Phys. Ther. Sci..

[43] Van Breukelen GJP (2006). ANCOVA versus change from baseline had more power in randomized studies and more bias in nonrandomized studies. J. Clin. Epid..

[44] Vincent WJ, Weir JP (2012). Statistics in Kinesiology.

[45] Cohen J (1992). A power primer. Psychol. Bull..

[46] Borisut S, Vongsirinavarat M, Vachalathiti R, Sakulsriprasert P (2013). Effects of strength and endurance training of superficial and deep neck muscles on muscle activities and pain levels of females with chronic neck pain. Phys. Ther. Sci..

[47] Lee S, Lee Y, Chung Y (2017). Effect of changes in head postures during use of laptops on muscle activity of the neck and trunk. Phys. Ther. Rehab. Sci..

[48] Thigpen CA, Lynch SS, Mihalik JP, Prentice WE, Padua D (2010). The effects of an exercise intervention on forward head and rounded shoulder postures in elite swimmers. Br. J. Sports Med..

[49] Kim SY, Kim NS, Laurentius JK (2015). Effects of cervical sustained natural apophyseal glide on forward head posture and respiratory function. Phys. Ther. Sci..

[50] Rondoni A, Rossettini G, Ristori D, Gallo F, Strobe M, Giaretta F, Battistin A, Testa M (2017). Intrarater and inter-rater reliability of active cervical range of motion in patients with nonspecific neck pain measured with technological and common use devices: A systematic review with meta-regression. J. Mani. Phys. Ther..

[51] Rossettini G, Testa M (2018). Manual therapy RCTs: Should we control placebo in placebo control?. Eur. J. Phys. Rehabil. Med..

