# Supplementary material for: Therapeutic routine with respiratory exercises improves posture, muscle activity, and respiratory pattern of patients with neck pain: a randomized controlled trial
Source: Sci Rep. 2022 Mar 9;12:4149. doi: 10.1038/s41598-022-08128-w (PMC8907211; doi:10.1038/s41598-022-08128-w)
Supplement: Supplementary file 1 — Supplementary Information 1. [file 41598_2022_8128_MOESM1_ESM.pdf]

Appendix 1

EMG electrodes were placed in five areas as follows: upper trapezius, as positioned from the lateral to the midpoint, as an imaginary line was formed by the posterior aspect of the acromion and the spinous process of C7, and the electrode was placed on the muscle bulk [23]. For the sternocleidomastoid, the electrode was placed at the lower one-third of the line connecting the sternal notch and mastoid process [23]. Forward head posture was defined as a cervical angle<50° [15-17]. A lateral-view photograph was taken to identify cervical angles in a standing position [15-17]. For scalene muscles, the electrode was placed on the posterior triangle of the scalene muscle, above the clavicle, more inclined to the sternocleidomastoid (just posterior to and at a slightly oblique angle relative to the sternocleidomastoid [SCM], just above the clavicle and in the hollow triangle anterior of the upper trapezius) [24]. For neck erector spine, the electrode was attached to the muscles around the C4 vertebra [25]. The diaphragm, lower edge of the rib cage on a vertical line that passes through the nipple centre was selected for electrode placement [26,27].

Appendix 2

Additionally, to estimate a maximum voluntary contraction (MVC) for the upper trapezius, subjects placed their hand at 90^0 abduction, sitting down on a chair, and were asked to apply pressure against the exposed resistance at the top [29]. To obtain the MVC for sternocleidomastoid and scalene muscles, subjects were placed in the supine position, and their hands were put on their own heads. Then, the head was anterolaterally placed and pressurised against the hand resistance [24]. To obtain the MVC for the erector spinae muscle, the subjects were asked to be in a prone posture and put both hands behind their head as moving the overhand against the resistance in the extension direction [29]. To achieve MVC for the diaphragm muscle in the sitting position subjects took deep breaths [30]. Each position of the maximal voluntary contraction was used two times for 5-second duration to normalise the data [29].

Appendix 3

Sit behind the subject and place both your hands on the lower lateral rib cage so that your whole hand rests firmly and comfortably and does not restrict breathing motion. Your thumbs should be approximately parallel to the spine, pointing vertically and your hand comfortably open with fingers spread so that the little finger approaches a horizontal orientation. Note that the 4th and 5th finger reach below the lower ribs and can feel abdominal expansion. You will make an assessment of the extent of overall vertical motion your hands feel relative to the overall lateral motion. Also decide if the motion is predominantly upper rib cage, lower rib cage/abdomen or relatively balanced. Use this information to determine the relative distance from the horizontal line (C) of the upper and lower lines of the MARM diagram. The upper line (A) will be further from the horizontal and closer to the top if there is more vertical and upper rib cage motion. The lower line (B) will be further from the horizontal and closer to the bottom if there is more lateral and lower rib cage/abdomen motion.[18] Finally get a sense of the overall magnitude and freedom of rib cage motion. Place lines further apart to represent greater overall motion and closer for less motion. the tester recorded and interpreted different aspects of respiration, including the number of breaths and the balance of respiration between the upper and lower parts of the rib cage and abdomen.[18]
