# Supplementary material for: Therapeutic routine with respiratory exercises improves posture, muscle activity, and respiratory pattern of patients with neck pain: a randomized controlled trial
Source: Sci Rep. 2022 Mar 9;12:4149. doi: 10.1038/s41598-022-08128-w (PMC8907211; doi:10.1038/s41598-022-08128-w)
Supplement: Supplementary file 3 — Supplementary Information 3. [file 41598_2022_8128_MOESM3_ESM.doc]

**Research design**

### Project summary

Neck pain and forward head posture are common in prolonged smartphone users and need to be targeted for treatment. We aimed to compare the effect of adding respiratory exercises to the therapeutic routine in smartphone users with forward-head posture and non-specific chronic neck pain. Sixty patients (aged 24.7±2.1 years) with forward-head posture and non-specific chronic neck pain were randomly assigned to the therapeutic routine (n=20), combined respiratory-exercises with therapeutic routine (n=20), or control groups (n=20) that that there was no difference among groups at pain intensity. Each programme was implemented three times a week for 8 weeks. Primary Outcomes Measures was pain, and secondary was forward head angle, electromyographic activity, and respiratory patterns that were measured at baseline and at 8-weeks’ post-treatment. There were significant difference in the combined group compared with the therapeutic routine group (p=0.03) for diaphragm muscle activation, respiratory balance (p=0.02), and number of breaths (p=0.02). There were significant within-group changes from baseline to post-treatment in the combined group for all outcomes above, but no changes in the therapeutic exercise routine group. Combination treatment is more effective than the standard therapeutic routine. These results could be strengthened by more studies with longer follow-up assessments.

### General information

Protocol title, protocol identifying number (if any), and date: **Effects of adding respiratory exercises to the therapeutic routine in smartphone users with forward head posture and non-specific chronic neck pain: a randomised controlled trial**

- Current Controlled Trials using the [IRCT](https://fa.irct.ir/) website with ID number of, IRCT20200212046469N1 “Prospectively registered” at 04/03/2020.
- Name and address of the sponsor/funder: None.
- Name and title of the investigator(s) who is (are) responsible for conducting the research, and the address and telephone number(s) of the research site(s), including responsibilities of each

Amir Letafatkar, PhD., Faculty of Physical Education and Sport sciences, Department of Biomechanics and Sport injuries, Kharazmi University, Tehran-Republic of Iran

Address: Biomechanics and Corrective Exercise Laboratory, Faculty of Physical Education and Sport sciences, Kharazmi University, Mirdamad Blvd., Hesari St, Tehran, Iran. Postal code: 00982122258084.

TEL: +989195394692 - +982634207184

E-mail Address: letafatkaramir[@yahoo.com](mailto:.hadadnezhad@yahoo.com)

AL, MH, HR, AP contributed to the original idea, study design and protocol, the conception of the work, conducting the study, data analysis, revising the drafting and editing of the article. AT, and AP contributed to the conception of the work, wrote, and editing of this article. All authors approved the final version of the article.

- Name(s) and address(es) of the clinical laboratory(ies) and other medical and/or technical department(s) and/or institutions involved in the research.

| Kharazmi University |  |
| --- | --- |
| **Division name** | department of biomechanics and sport injuries |
| **Zip code** | 4352763633 |
| **Address** | tehran-mirdamad-sout razan street, faculty of PE |
| **TEL** | 009802122258084 |

### Rationale & background information

The use of electronic tools is increasing worldwide [1]. Varieties and the attractiveness of these tools have led to various groups of people, especially teenagers, using them for prolong periods and this has increased some related musculoskeletal problems [2].

The weight of an adult's head in neutral position is 10-12 pounds. There is an increase in load towards the neck with the flexion of the head and the weight is calculated to be 27, 40, 49 and 60 pounds at 15°, 30°,45° and 60° respectively [3]. When using a smartphone, people usually have a downward gaze to stare at the lowered objects and maintain the head in a forward position for long periods of time which may cause neck pain. Moreover, the maintenance of a forward head posture (FHP) decrease cervical lordosis of the lower cervical vertebrae and creates a posterior curve in the upper thoracic vertebrae to maintain balance. This is known as FHP [3]. It is commonly thought that in addition to psychological problems, like anxiety, headaches, insomnia, depression, poor sleep quality, and fatigue, long-term use of smartphones leads to inactivity and abnormal posture, such as the FHP and rounded shoulders [4]. This can necessarily affect muscular activity, and place more pressure on the cervical spine where chronic pain originates [5,6].

Individuals with FHP and chronic neck pain often suffer from weakness of the deep neck flexor muscles, which is to be compensated by excessive activity of other muscles such as sternocleidomastoid, and scalene [7]. This compensation leads to muscle imbalance and changes in the stress-strain diagram, by which cervical spine overload occurs [7,9]. This may also be observed in the thoracic spine as some involved muscles are connected to both areas. Also, disorders or impairments occur in the neck and respiratory system because of the joint activity of the above-mentioned muscles operated on the neck movements and respiratory function [7,8].

Maximal inspiratory and expiratory pressure (MIP & MEP) decrease in individuals with chronic neck pain and FHP [8]. This situation causes the respiratory pattern to change from nasal breathing to mouth breathing. As compared to the normal posture, both scalene and sternocleidomastoid muscles show a higher activity amount in FHP [7]. Such long-term activation can create poor respiratory habits to facilitate activities in auxiliary respiratory muscles [5]. Furthermore, the respiratory function is affected by changed muscle activity due to pain and disability, affecting the neck in a vicious circle. In order to improve this posture, heat, traction, and exercise have all been used. Various methods such as joint mobilization, stretching, isometric strengthening exercises, endurance exercises, and proprioceptive exercises have also been applied depending on the method and theory utilized by the therapist or the patient’s condition [9]. Also in some previous researches various therapeutic and rehabilitative methods have been used such as the McKenzie exercise, Kinesio taping, and myofascial release [10]. Each method has demonstrated positive results in improving impairments and disorders in this area [10]. In this regard, some researchers have reported better consequences obtained from combinations of some of their therapeutic methods [10]. But previous research has not compared the effect of adding breathing exercises to therapeutic exercises; also respiratory exercises have shown benefits for respiration and balancing the main and auxiliary muscles [7]. Respiratory exercises are low cost and are also easily used in different situations [7]. In this regard, the present research aimed to compare the effect of adding respiratory exercises to the therapeutic routine on pain, electromyography (EMG), posture, and respiratory patterns in smartphone users with FHP and non-specific chronic neck pain. We hypothesised that adding respiratory exercise would enhance treatment effects on neck pain, respiratory pattern, electromyography, and posture in smartphone users with moderate chronic neck pain and FHP.

### References (of literature cited in preceding sections)

1. Lee NK, Jung SI, Lee DY, Kang KW. Effects of exercise on cervical angle and respiratory function in smartphone users. Oso Pub Heal Rese. Persp. 2017; 8: 271-4.

###

### Study goals and objectives

### The purpose of our study was to examine the effects of adding respiratory exercises to the therapeutic routine in smartphone users with forward head posture and non-specific chronic neck pain.

### Study design and Methodology

The study was a randomised assessor-blind controlled trial using the [IRCT](https://fa.irct.ir/) website with ID number of, IRCT20200212046469N1 “Prospectively registered” at 04/03/2020. Patients with chronic neck pain were recruited by orthopaedic physicians via flyers displayed at the hospitals over 3-months from April to June 2020.

Prior to participation at the study, all subjects were explained about the objectives and provided written informed consent and all participants provided written informed consent prior to enrolment. This study was performed in accordance with the 1964 Helsinki declaration, its later amendments and local ethics committee by the Research Ethics Committee of the Kharazmi University.

Inclusion criteria were males and females who using a smartphone for more than 4=hours a day who rated their ‘worst pain over the last 24-hours’ as moderate using the visual analogue scale (VAS), with neck disability index (NDI) scores between 28% and 45%, and pain lasting longer than 3 months [11-14]. FHP was defined as a cervical angle <50° [15-17]. A lateral-view photograph was taken to identify cervical angle in standing position [15-17].

The exclusion criteria were, previous history of neck or back surgery, neurological signs, rheumatoid arthritis, and currently using muscle relaxation medication. The local ethics committee at University approved the study protocol and all subjects signed informed consent.

Participants were randomised into two experimental groups and one control group, by drawing a number, from 1 to 63, placed in sealed envelopes in a box prepared in advance by the trainer. The randomisation sequence was not disclosed until participants had completed their baseline assessments. The assessor was blinded to group allocation. Participants were not blinded to exercise study; however, they were not aware which treatment was considered to be therapeutic. The same physiotherapist and trainer supervised both active treatment groups.

### Safety considerations

No adverse event was reported.

### Follow-up

Not applicable.

### Data management and statistical analysis

The necessary sample size was estimated using G*Power 3.1.7 for Windows (G*Power©, University of Dusseldorf, Germany). To obtain 80% statistical power, an α error =0.05, repeated-measure analysis of variance (ANOVA), and a medium effect size of 0.25 to consider two groups and two measurements for the primary outcome (neck pain), generating a sample size about of 18-participants per group (total sample size of 54-subjects) considering a 15%-dropout rate the sample was increased to 63 (21 in each group).

One-way ANOVA was used to compare the group demographics. The analysis of covariance (ANCOVA), with a between-factor of the groups and participants' baseline scores included as a covariate [39]. The Bryant-Paulson procedure was used when conducting the pairwise comparisons and calculating the confidence intervals [40]. Effect sizes of 0.2, 0.5, and 0.8 were considered ‘small’, ‘moderate’, and ‘large’ respectively [41]. SPSS software was (alpha level of .05) used for statistical analysis (IBM Corp., Armonk, NY, USA).

### Quality assurance

Quality of data’s checked by authors.

### Expected outcomes of the study

We expected that athletes who participated in neuromuscular training would exhibit improvements in biomechanics, knee proprioception, and function that exceed those exhibited by athletes who simply continued their typical training routine.

### Dissemination of results and publication policy

Not applicable.

### Duration of the project

Expected recruitment start date

2020-03-10, 1398/12/20

Expected recruitment end date

2020-05-09, 1399/02/20

Actual recruitment start date

2020-03-10, 1398/12/20

Actual recruitment end date

2020-05-09, 1399/02/20

Trial completion date

2020-08-10, 1399/05/20

### Problems anticipated

Not applicable.

### Project management

AL, MGH, TA, SK contributed to the original idea, study design and protocol, the conception of the work, conducting the study, data analysis, revising the drafting and editing of the article. AT, and AL contributed to the conception of the work, wrote, and editing of this article. All authors approved the final version of the article.

### Ethics

This study was performed in accordance with the 1964 Helsinki declaration, its later amendments and local ethics committee. This study was approved by an Akhtar Orthopedic Hospital Institutional Review Board in human subjects.

### Informed consent forms

Prior to participation at the study, all subjects were explained about the objectives and provided written informed consent and all participants provided written informed consent prior to enrollment.

#### Budget

None.

#### Other support for the project

None.

#### Collaboration with other scientists or research institutions:

#### None.

#### Links to other projects:

#### none.

#### Curriculum Vitae of investigators

Hamid Rezaee Dareh-deh^1^

Malihe Hadadnezhad^2^

Amir Letafatkar^3^

Anneli Peolsson^4^

1. MS, Faculty of Physical Education and Sports Sciences, Kharazmi University, Tehran, IRN, E-mail: h.rezaee8858@gmail.com
2. PhD, Assistant Professor, Sport Injury and Corrective Exercises, Kharazmi University, Tehran, IRN, E-mail: m.hadadnezhad@yahoo.com

3. PhD, Assistant Professor, Sport Injury and Corrective Exercises, Kharazmi University, Tehran, IRN, E-mail: letafatkaramir@yahoo.com

4. Professor, PhD, MSc Physiotherapist, Department of Health, Medicine and Caring Sciences, Unit of Physiotherapy, [Linköping University](https://www.researchgate.net/institution/Linkoeping_University), Linköping, Sweden, E-mail: Anneli.Peolsson@liu.se

#### Other research activities of the investigators

Note involved.

#### Financing and insurance

None.
