# Supplementary material for: Therapeutic routine with respiratory exercises improves posture, muscle activity, and respiratory pattern of patients with neck pain: a randomized controlled trial
Source: Sci Rep. 2022 Mar 9;12:4149. doi: 10.1038/s41598-022-08128-w (PMC8907211; doi:10.1038/s41598-022-08128-w)
Supplement: Supplementary file 4 — Supplementary Information 4. [file 41598_2022_8128_MOESM4_ESM.pdf]

Clinical Trial Protocol
Iranian Registry of Clinical Trials

18 Sep 2021

Comparison the addition of respiratory exercises to corrective exercises on pain, electromyography, posture, and respiratory pattern in Smartphone users with forward head posture and non-specific chronic neck pain

Protocol summary

Study aim

Comparison the addition of respiratory exercises to corrective exercises on pain, electromyography, posture, and respiratory pattern in Smartphone users with forward head posture and non-specific chronic neck pain

Design

Randomized control trial, With parallel groups, Double blind, randomized

Settings and conduct

60 subjects with forward head posture and Non-specific chronic neck pain that met the inclusion criteria was randomly divided into three intervention groups (Corrective _ Respiratory Exercise Group, Corrective Exercise Group and Control Group). Groups Training interventions will be performed 3 sessions per week for 8 weeks.

Participants/Inclusion and exclusion criteria

Inclusion criteria: 20-30 year old students, 2 to 4 hours of smartphone use per day, With non-specific chronic neck pain averaging between 3 and 7 out of 10 on the Visual Analog Scale (VAS) scale in the past 3 months, Having a forward head and shoulder posture less than 50 degrees, body Math Index (BMI) between 19 and 25. Exclusion criteria: History of bone fracture, Neuromyopathy, Inflammation or inflammation of the spinal cord, History of spinal cord injury and surgery, Cardiovascular disease, Trauma, Hypertension, Respiratory disorders, smoking habit, Nerve deficiency,Poor public health, Chronic lung disease, MS.

Intervention groups

Combining Corrective Exercises with Respiratory Exercises 2. Corrective Exercises 3. Daily Activities

Main outcome variables

Pain,Posture of head and neck, Electromyography of superficial neck muscles (upper trapezius, sternocleidomastoid, scalene, diaphragm and spine neck erector) and Respiratory pattern

General information

Reason for update

To enter the start and end dates of the disease

Acronym

IRCT registration information

IRCT registration number: **IRCT20200212046469N1**
Registration date: **2020-03-04, 1398/12/14**
Registration timing: **prospective**

Last update: **2020-10-18, 1399/07/27**
Update count: **4**

Registration date

2020-03-04, 1398/12/14

Registrant information

Name

Hamid Rezaee dareh deh

Name of organization / entity

Center for Human Movement Sciences Kharazmi University

Country

Iran (Islamic Republic of)

Phone

+98 21 2222 8001

Email address

std_h.rezaee@khu.ac.ir

Recruitment status

Recruitment complete

Funding source

Expected recruitment start date

2020-03-10, 1398/12/20

Expected recruitment end date

2020-05-09, 1399/02/20

Actual recruitment start date

2020-03-10, 1398/12/20

Actual recruitment end date

2020-05-09, 1399/02/20

Trial completion date

2020-08-10, 1399/05/20

Scientific title

Comparison the addition of respiratory exercises to corrective exercises on pain, electromyography, posture, and respiratory pattern in Smartphone users with forward head posture and non-specific chronic neck pain

Public title

Comparison the addition of respiratory exercises to corrective exercises on pain, electromyography, posture, and respiratory pattern in Smartphone users with forward head posture and non-specific chronic neck pain

Purpose

Treatment

Inclusion/Exclusion criteria

Inclusion criteria:

20-30 year old students 2 to 4 hours of smartphone use per day Non-specific chronic neck pain ranging from 3 to 7 out of 10 on the VAS scale in the past 3 months Having a forward head and shoulder posture with an angle of less than 50 degrees BMI between 19 and 25

Exclusion criteria:

History of bone fractures Neuromyopathy Infection or inflammatory arthritis in the cervical spine History of spinal cord injury and surgery trauma Hypertension Respiratory disorders Smoking habit Nerve defect Poor public health Chronic pulmonary disease MS Cardiovascular diseases

Age

From **20 years** old to **30 years** old

Gender

Both

Phase

N/A

Groups that have been masked

- Investigator
- Outcome assessor
- Data analyser

Sample size

Target sample size: **60**

Actual sample size reached: **60**

Randomization (investigator's opinion)

Randomized

Randomization description

Balanced block randomization

Blinding (investigator's opinion)

Double blinded

Blinding description

The outcome evaluator, participant, researcher, and data analyst are kept blind.

Placebo

Not used

Assignment

Parallel

Other design features

Secondary Ids

empty

Ethics committees

1

Ethics committee

Name of ethics committee

Ethics committee of Kharazmi University

Street address

Center for Human Movement Sciences Kharazmi University Mirdamad, South Razan Street, Hesari Street, Keshvari Sport Complex, Tehran, Iran

City

Tehran

Province

Tehran

Postal code

15447-33111

Approval date

2019-09-30, 1398/07/08

Ethics committee reference number

IR.KHU.REC.1398.023

Health conditions studied

1

Description of health condition studied

Forward head posture

ICD-10 code

R29.3

ICD-10 code description

Abnormal posture

2

Description of health condition studied

Chronic neck pain

ICD-10 code

G89.29

ICD-10 code description

Other chronic pain

Primary outcomes

1

Description

Pain

Timepoint

Before and after the intervention

Method of measurement

VAS

2

Description

Forward head angle
Timepoint
Before and after the intervention
Method of measurement
Photogrammetry

Secondary outcomes

1

Description
Muscle Activity Level 1- Upper Trapezius 2- Sternocleidomastoid 3- Scalene 4- Neck Erector Spine 5- Diaphragm
Timepoint
Before and after the intervention
Method of measurement
Electromyography device

2

Description
Onset of muscle activity 1. Upper trapezius, 2. Sternocleidomastoid 3. Scalene 4. neck erector Spine 5. Diaphragm
Timepoint
Before and after the intervention
Method of measurement
Electromyography device

3

Description
Respiratory pattern
Timepoint
Before and after the intervention
Method of measurement
Manual Assessment of Respiratory Motion (MARM)

Intervention groups

1

Description
Intervention group: combination of corrective exercises and respiratory exercises, One session a day, three sessions a week, for eight weeks
Category
Rehabilitation

2

Description
Intervention group: Corrective Exercises, One session a day, three sessions a week, for eight weeks
Category
Rehabilitation

3

Description
Control group: Doing daily activities
Category
Rehabilitation

Recruitment centers

1

Recruitment center
Name of recruitment center
Kharazmi University Health and Wellness Center
Full name of responsible person
Hamid Rezaee darehdeh
Street address
Center for Human Movement Sciences Kharazmi University Mirdamad, Sout Razan Street, Hesari Street, Keshvari Sport Complex, Tehran, Iran
City
Tehran
Province
Tehran
Postal code
15447-33111
Phone
+98 21 2222 8001
Fax
Email
std-h.rezaee@khu.ac.ir
Web page address

Sponsors / Funding sources

1

Sponsor
Name of organization / entity
Kharazmi University
Full name of responsible person
Amir letafatkar
Street address
-
City
Tehran
Province
Tehran
Postal code
-
Phone
Fax
Email
letafatkaramir@yahoo.com
Grant name
Grant code / Reference number
Is the source of funding the same sponsor organization/entity?
Yes
Title of funding source
Kharazmi University
Proportion provided by this source
20
Public or private sector
Public
Domestic or foreign origin

Domestic
Category of foreign source of funding
empty
Country of origin
Type of organization providing the funding
Academic

Person responsible for general inquiries

Contact
Name of organization / entity
Center for Human Movement Sciences Kharazmi University
Full name of responsible person
Hamid Rezaee darehdeh
Position
Master student of Corrective Exercises and Sport injury prevention
Latest degree
Master
Other areas of specialty/work
Corrective Exercises and Sport injury prevention
Street address
Center for Human Movement Sciences Kharazmi University Mirdamad, Sout Razan Street, Hesari Street, Keshvari Sport Complex, Tehran, Iran
City
Tehran
Province
Tehran
Postal code
15447-33111
Phone
+98 21 2222 8001
Email
std-h.rezaee@khu.ac.ir

Person responsible for scientific inquiries

Contact
Name of organization / entity
Center for Human Movement Sciences Kharazmi University
Full name of responsible person
Hamid Rezaee darehdeh
Position
Master student of Corrective Exercises and Sport injury prevention
Latest degree
Master
Other areas of specialty/work
Corrective Exercises and Sport injury prevention
Street address
Center for Human Movement Sciences Kharazmi University Mirdamad, Sout Razan Street, Hesari Street, Keshvari Sport Complex, Tehran, Iran
City
Tehran
Province
Tehran
Postal code
15447-33111
Phone
+98 21 2222 8001
Email
std-h.rezaee@khu.ac.ir

Person responsible for updating data

Contact
Name of organization / entity
Center for Human Movement Sciences Kharazmi University
Full name of responsible person
Hamid Rezaee darehdeh
Position
Master student of Corrective Exercises and Sport injury prevention
Latest degree
Master
Other areas of specialty/work
Corrective Exercises and Sport injury prevention
Street address
Center for Human Movement Sciences Kharazmi University Mirdamad, Sout Razan Street, Hesari Street, Keshvari Sport Complex, Tehran, Iran
City
Tehran
Province
Tehran
Postal code
15447-33111
Phone
+98 21 2222 8001
Email
std-h.rezaee@khu.ac.ir

Sharing plan

Deidentified Individual Participant Data Set (IPD)
Yes - There is a plan to make this available
Study Protocol
Yes - There is a plan to make this available
Statistical Analysis Plan
Yes - There is a plan to make this available
Informed Consent Form
Yes - There is a plan to make this available
Clinical Study Report
Yes - There is a plan to make this available
Analytic Code
Yes - There is a plan to make this available
Data Dictionary
Yes - There is a plan to make this available
Title and more details about the data/document
Only part of the data, such as the dependent variables, The average of all samples, can be shared in scientific articles.
When the data will become available and for how long
The date of access is August 2020.
To whom data/document is available
Personal information is confidential and general results are available to anyone in the article.

Under which criteria data/document could be used

Information is not available to anyone. general results are available to anyone in the article.

From where data/document is obtainable

Hamid Rezaee darehdeh 0098 9353872375 - Email: std-h.rezaee@khu.ac.ir

What processes are involved for a request to access data/document

-

Comments

-
